# Supplementary material for: Factor analysis of multimodal MRI, biofluid, and vascular biomarkers reveals latent constructs of brain health
Source: GeroScience. 2025 Jul 8;48(2):1885–903. doi: 10.1007/s11357-025-01771-x (PMC12972403; doi:10.1007/s11357-025-01771-x)
Supplement: Supplementary file 1 — Supplementary file1 (DOCX 537 KB) [file 11357_2025_1771_MOESM1_ESM.docx]

Factor Analysis of Multimodal MRI, Biofluid and Vascular Biomarkers Reveals Latent Constructs of Brain Health

Ella Rowsthorn^a,b^, Ming Ann Sim^b,c,d^, William T. O’Brien^a^, Stuart J. McDonald^a^, Katherine Franks^b^, Benjamin Sinclair^a^, Trevor T.-J. Chong^b,e,f^, Stephanie Yiallourou^b^, Marina Cavuoto^b,g^, Lucy Vivash^a^, Terence J. O’Brien^a^, Xingfeng Shao^h^, Danny J.J. Wang ^h^, Meng Law^a,i,j^, Ian H. Harding^a,k*^, Matthew P. Pase^b*#^

*These authors contributed equally.
^#^Corresponding author.

1. Department of Neuroscience, School of Translational Medicine, Monash University, 99 Commercial Road, Melbourne, VIC 3004, Australia
2. School of Psychological Sciences and Turner Institute for Brain and Mental Health, Monash University, 18 Innovation Walk, Clayton, VIC 3168, Australia
3. National University Health System, National University Hospital, 1E Kent Ridge Rd, Kent Ridge, 119228, Singapore
4. Yong Loo Lin School of Medicine, National University of Singapore, 10 Medical Dr, Kent Ridge, 117597, Singapore
5. Department of Neurology, Alfred Health, 55 Commercial Road, Melbourne, VIC 3004, Australia
6. Department of Clinical Neurosciences, St Vincent’s Hospital, 41 Victoria Parade, Fitzroy, VIC 3065, Australia
7. National Ageing Research Institute, The Royal Melbourne Hospital, 34-54 Poplar Road, Parkville, VIC 3052, Australia
8. Laboratory of FMRI Technology (LOFT), Stevens Neuroimaging and Informatics Institute, University of Southern California, Los Angeles, CA 90033, USA
9. Department of Radiology, Alfred Health, 99 Commercial Road, Melbourne, VIC 3004, Australia
10. Department of Electrical and Computer Systems Engineering, Monash University, 14 Alliance Lane, Clayton, VIC 3168, Australia
11. QIMR Berghofer Medical Research Institute, 300 Herston Rd, Herston, QLD 4006, Australia

**Correspondence:**

Assoc/Prof. Matthew P. Pase

School of Psychological Sciences
Monash University
18 Innovation Walk
Clayton VIC 3168
Australia

E: matthew.pase@monash.edu

**Table of Contents**

MRI Quality Control (QC) and Exclusion Criteria 4

Principal Components Analysis of Cognitive Tests 7

Exploratory Factor Analysis (EFA) Scree Plots 10

Basal Ganglia ePVS Post-hoc Analysis 11

Correlations between Construct Composites 13

WM Fluid Dysregulation Post-hoc Analysis 14

Relationships between Construct Composites and Individual Cognitive Tests 15

References 16

**MRI Quality Control (QC) and Exclusion Criteria**

All raw MRI data and derived outcomes underwent quality control (QC) by ER, including both visual inspection and automated assessment using the MRIQC software package^1^ (v0.14.2).

Structural Imaging (PVS and WMH)

Scans were visually inspected for common artifacts such as field inhomogeneities, motion-related distortion, motion distortion and poor signal-to-noise ratio (SNR). Quantitative metrics, including contrast to noise (CNR), percentage of artifact voxels (QI1), smoothness (FWHM), foreground-to-background energy ratio (FBER), or entropy focus criterion (EFC) and SNR, were extracted with MRIQC. Scans with metrics exceeding 1.5 times above or below the interquartile range (IQR) of the cohort distribution were flagged as ‘high risk’ for segmentation failure. These were subsequently excluded if visual inspection confirmed compromised tissue contrast and segmentation accuracy (e.g. motion distortion misclassified as perivascular on T1w images).

Diffusion (Free Water and fwFA)

Raw DWI images were inspected for motion artifacts, including for the “venetian blind” effect. If less than 10% of the 127 volumes in a timeseries exhibited severe motion, these volumes were removed prior to QSIPrep^2^ (version 0.14.3, based on Nipype 1.6.1) processing, provided sufficient remaining data were available in each b-value category. Scans with >10% affected volumes were excluded. Pre-processed images and QSIPrep reports were reviewed, including inspection of b0 identification and averaging, quality of susceptibility distortion correction and field displacement severity across the timeseries.

pCASL (CBF)

M0 images were treated as pseudo-T2w images for QC purposes. MRIQC metrics (CNR, QI1, FWHM, FBER, EFC and SNR) were evaluated, and scans falling outside 1.5 times IQR threshold were flagged as ‘high risk’. Visual inspection assessed motion artifacts and placement of acquisition ‘slab’ to ensure whole-brain coverage. Mean grey matter (GM) and white matter (WM) cerebral blood flow (CBF) values were calculated for each CBF map; scans with values outside of physiologically plausible ranges (GM: 30-90 mL/100g/min)^3^ were excluded due to suspected processing failure.

DP-pCASL/BBB k_w_ QC

After reconstruction and processing via the LOFT Toolbox^4^, blood-brain barrier water exchange rate (BBB k_w_) maps and associated M0 images were visually inspected for motion blur, field inhomogeneity and placement of acquisition ‘slab’. Mean whole-brain BBB k_w_ values were computed, and maps with implausible values (<40 or >180 min^-1^)^5,6^ were excluded.

Registration QC

All automated image registrations to T1w space were visually confirmed for accuracy, including for WMH, Free Water, fwFA and CBF.

Final MRI Sample

Of the 149 participants available for analysis, 17 were excluded due to incomplete or poor-quality MRI scans: 3 participants with motion distortion and poor SNR leading to inaccurate PVS segmentation; 1 participant with poor SNR affecting WMH segmentation; 1 with tissue misclassification and partial volume effects affecting CBF analysis; 3 with DP-pCASL issues including 1 with erroneous data acquisition, 1 with inadequate acquisition ‘slab’ placement and 1 with abnormally low mean BBB k_w_; 3 with excessive motion affecting multiple sequences; and 6 who did not completed the full MRI protocol (4 partially incomplete, 2 without MRI).

**Principal Components Analysis of Cognitive Tests**

We conducted a principal components analysis to determine tests that best represented global cognitive function to form our primary cognition outcome variable. Firstly, we used parallel analysis scree plots to guide the number of factors that best suit the data, where a single-factor, two-factor, three-factor or four-factor models were deemed appropriate. Across all factor solutions, the first principal component (PC1) consistently emerged as a robust measure of global cognition, comprising seven of the nine cognitive outcomes. The four-factor model (**eTable 1**) produced the most optimal fit metrics, had high interpretability and was therefore retained for further analyses (model comparison in **eTable 2**).

To evaluate the internal consistency of the cognitive tests contributing to the global cognition factor (PC1), we calculated Cronbach’s alpha using the factor loadings form the four-factor PCA model. All cognitive tests that loaded strongly onto PC1 (>0.45) were included in the analysis. Cronbach’s alpha for PC1 was 0.77 (Feldt’s 95% CI = [0.70, 0.83]), and no single item would improve alpha if removed, suggesting internal consistency across all included tests.

|  | **PC1** | **PC2** | **PC3** | **PC4** |
| --- | --- | --- | --- | --- |
| **Prose Passages Delayed** | **0.50** | **0.49** | -0.32 | 0.32 |
| **Similarities** | **0.67** | 0.41 | -0.12 | 0.10 |
| **SYDBAT Naming** | **0.76** | 0.01 | **0.42** | -0.01 |
| **Visual Repro. Delayed** | **0.64** | 0.30 | 0.08 | 0.02 |
| **TMT A*** | -0.44 | **0.51** | **0.43** | 0.43 |
| **TMT B*** | **-0.72** | 0.15 | 0.33 | 0.06 |
| **Verbal Fluency** | **0.63** | 0.03 | 0.09 | -0.44 |
| **HVOT** | **0.53** | -0.41 | **0.59** | 0.15 |
| **TASIT** | 0.32 | **-0.61** | -0.24 | **-0.58** |

**Note:** *Lower values indicate better performance.
Loadings >0.45 were considered to strongly load onto a given factor.
SYDBAT = Sydney Language Battery ; Visual Repro. = Visual Reproduction; TMT = Trail Making Test; HVOT = Hooper Visual Organisation Test; TASIT = The Awareness of Social Inference Test.

**eTable 1. Principal Components Analysis of Cognitive Tests: Four-factor model loadings**

**eTable 2. Principal Components Analysis of Cognitive Tests: Model Comparison**

| **Model** | **RMSR** | **Chi-Square (χ^2^)** | **χ^2^ p-value** | **Variance Explained*** | **Mean Item Complexity** |
| --- | --- | --- | --- | --- | --- |
| One-factor | 0.12 | 127.60 | 5.4e^15^ | 83% | 1.0 |
| Two-factor | 0.11 | 117.64 | 3.1e^16^ | 85% | 1.5 |
| Three-factor | 0.10 | 91.29 | 2.8e^14^ | 88% | 1.9 |
| Four-factor | 0.09 | 71.23 | 2.3e^13^ | 91% | 2.4 |

**Note:** RMSR = root mean square of residuals.
*Percentage of variance explained reflects the off-diagonal variance in the correlation matrix.
N=127


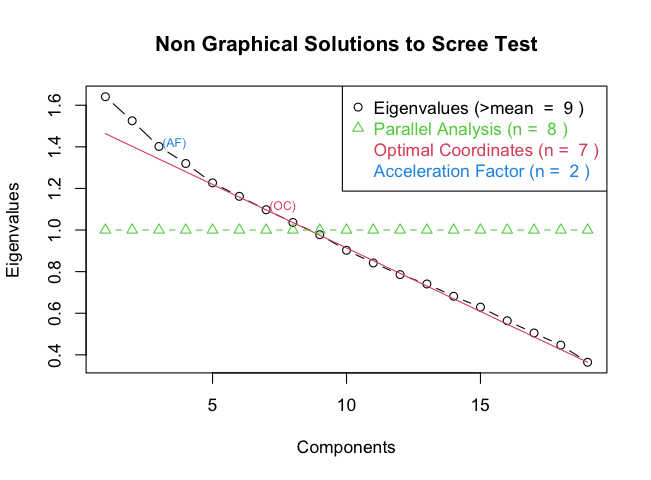

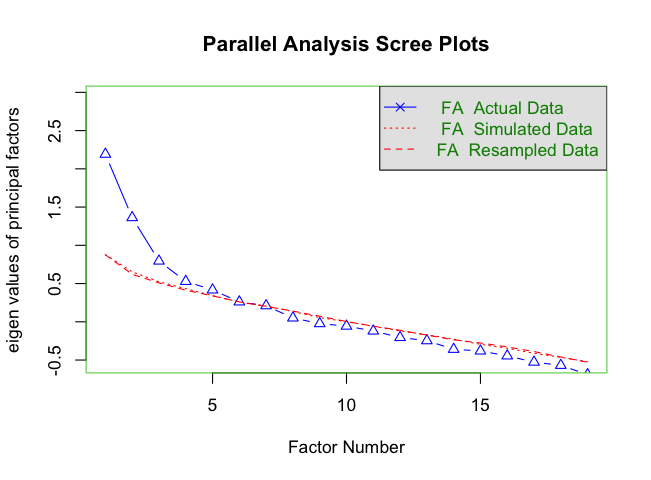


**1a.**

**1b.**

**eFigure 1: Exploratory Factor Analysis (EFA) Scree Plots**

Scree plots were used to determine the numbers of factors to retain. While the traditional ‘elbow’ method was less clear, five factors were retained as they met criterion below and maintained conceptual coherence.

**a.** **Non-Graphical Solutions to the Scree Test:** This plot compares multiple factor retention methods. Parallel analysis (green) suggested 8 factors, optimal coordinates (red) suggested 7, and the acceleration factor method (blue) suggested 2. A five-factor solution was supported as a balance between these methods and theoretical interpretability.

**b. Parallel Analysis Scree Plot:** This plot offers a robust refinement of the scree test findings. The retainment of n-factors was supported if their eigenvalues (blue) exceeded those of the simulated (dotted red) and resampled (dashed red) data. This method also supported a five-factor solution.

**Basal Ganglia ePVS Post-hoc Analysis**

We observed that greater basal ganglia (BG) enlarged perivascular space volume (ePVS) loaded onto the Brain & Vascular Health and Structural Health factors, which went against our expectations. To better understand this finding, we conducted post-hoc analyses, first exploring potential methodological bias. While we analysed ePVS as a volume fraction to minimise the effect of individual region volume variability, it is possible that those with larger brain volumes (or less atrophy) have a larger available area for ePVS to occur. Linear regressions adjusting for age, sex and intracranial volume confirmed a significant positive relationship between BG region volume and total BG ePVS volume (β=0.72, SE=0.11, *p*<.001, R^2^_adj_=.358), with a similar but attenuated effect for ePVS volume fraction (β=0.54, SE=0.12, *p*<.001, R^2^_adj_=.301). Interestingly, while previous cross-sectional studies report that ePVS volumes increase with age^7,8^, we found that BG ePVS volume fraction *decreased* with age across our cross-sectional cohort (β=-0.39, SE=0.08, *p*<.001, R^2^_adj_=.151). There was a significant interaction effect between ePVS and BG region volume, where BG ePVS volume fraction instead increased with age in those with the very largest BG region volumes within our data (interaction p=.010), but decreased with age in the remainder of the cohort (**eFigure 2**). This demonstrates that the pairing of BG ePVS with better health outcomes and the negative relationship between BG ePVS and age is not solely explained by region size effects. Although this does not completely rule out other potential methodological biases, it does suggest that our BG ePVS findings may have biological relevance.


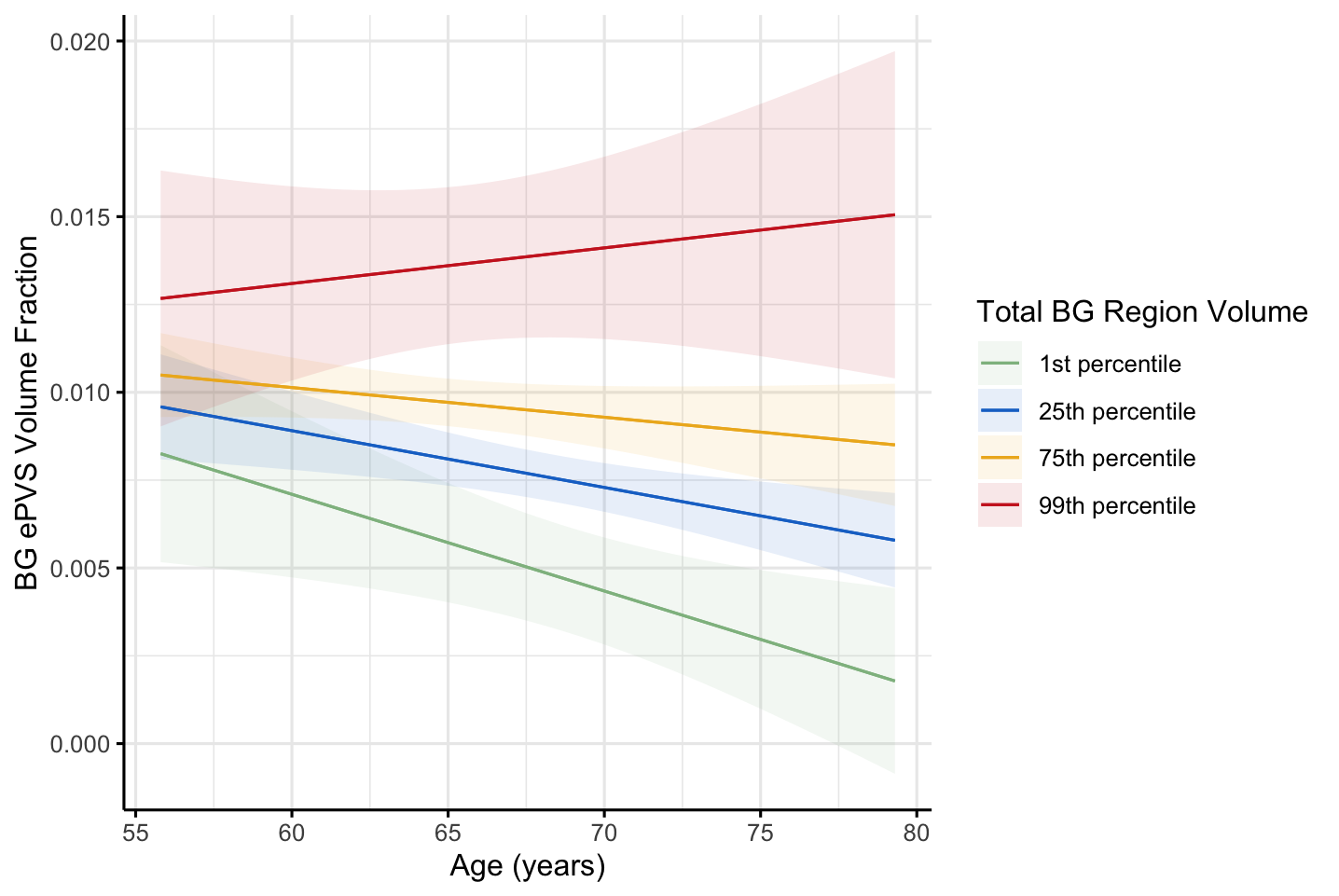


**eFigure 2: Interaction effect between BG region volume, BG ePVS volume fraction and age.**A linear regression model adjusting for sex and intracranial volume tested the interaction effect of total basal ganglia (BG) region volume on the association between BG enlarged perivascular space (ePVS) volume fraction and age.

Although there was a significant interaction effect, BG ePVS volume fraction increased with age in only those with the very largest BG region volumes within our data (at approximately the 99^th^ percentile) and decreased with age for the remainder of the cohort.


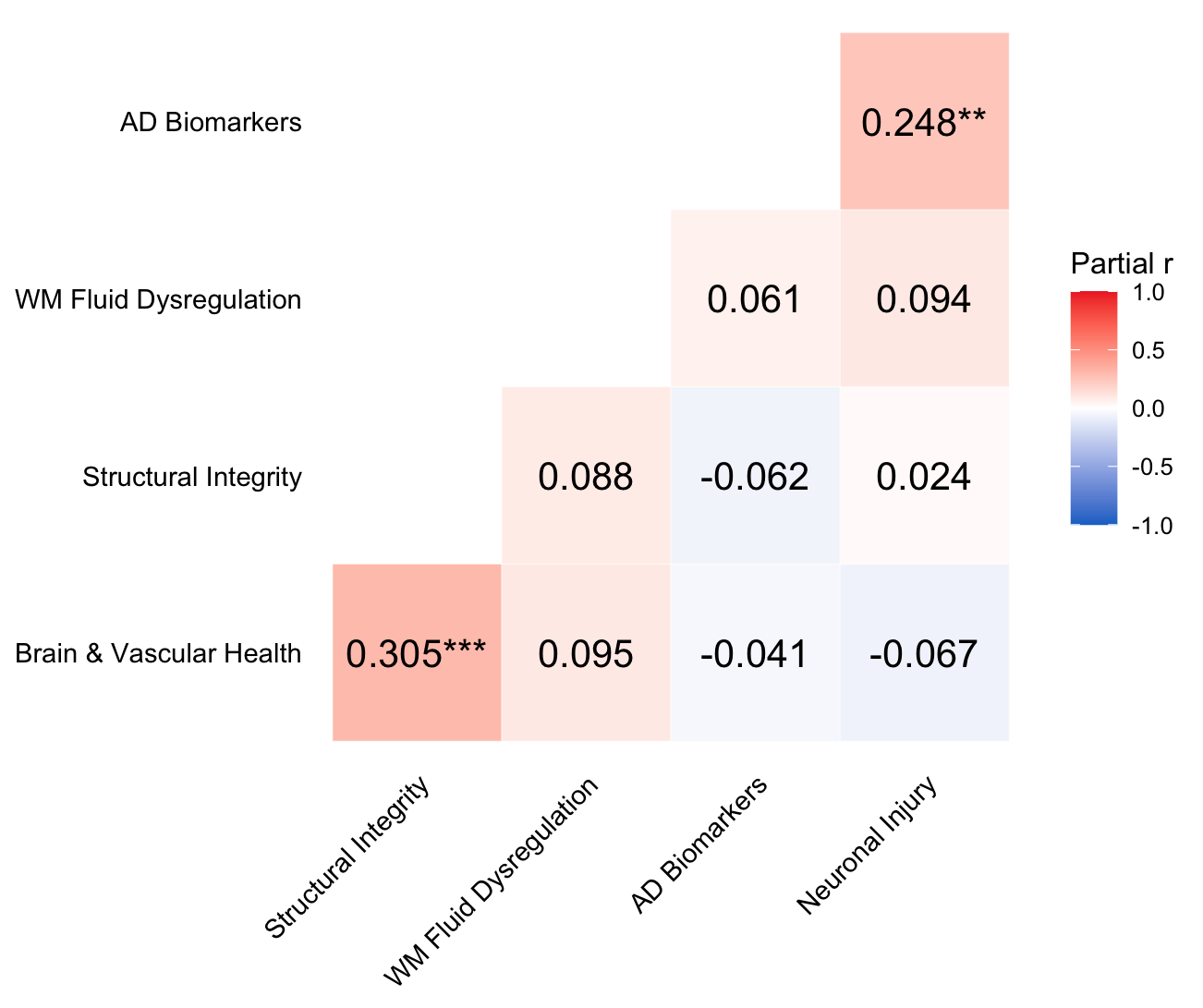


**eFigure 3. Correlations between Construct Composites**Pearson’s partial correlations between each of the construct composites, adjusting for sex and intracranial volume. AD = Alzheimer’s disease; WM = white matter.
***p*<.01, ****p*<.001.

**WM Fluid Dysregulation Post-hoc Analysis**

Although prior literature reports age-related increases in fluid dysregulation, we found no association between age and the WM Fluid Dysregulation construct. To better understand this null association, we examined each component individually using separate linear models adjusted for sex and intracranial volume. In agreeance with previous studies, WM Free Water volume fraction was positively associated with age (β=0.265, SE=0.084, p=.002; **eFigure 4a**). In contrast, WM ePVS volume fraction was negatively associated with age (β=-0.187, SE=0.084, p=.027; **eFigure 4b**). Therefore, when combined in a composite variable, these opposing associations may have contributed to the absence of an age relationship for the WM Fluid Dysregulation construct variable.


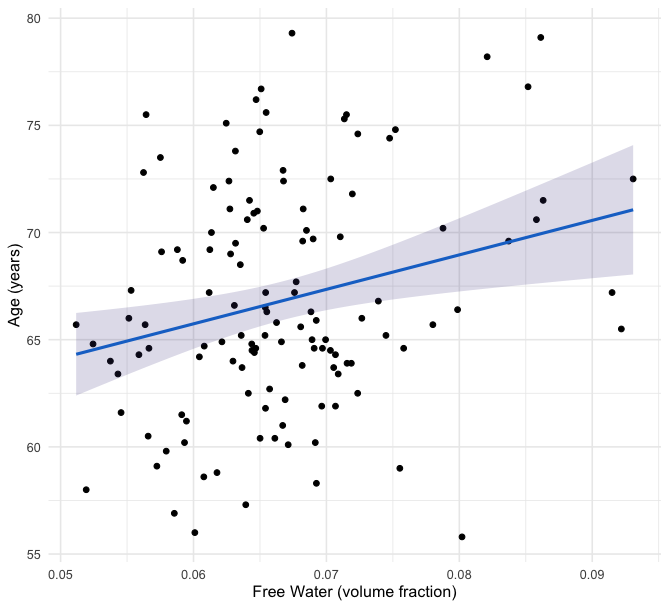

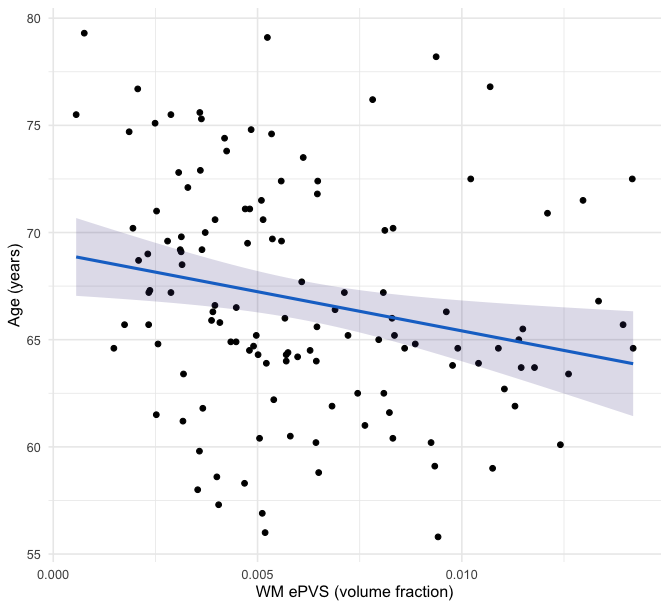


**4b.**

**4a.**

**eFigure 4. Associations between Age and Components of the WM Fluid Dysregulation Construct**Multiple linear regression adjusting for sex and intracranial volume.
a. Association between age and Free Water
b. Association between age and WM ePVS

WM = white matter.

**eTable 3. Relationships between Construct Composites and Individual Cognitive Tests**

**Note:** *Lower values indicate better performance.

Multiple linear regressions between each cognitive outcome and factor constructs. All models adjusted for age, sex, estimated intracranial volume and education. AD = Alzheimer’s disease; SYDBAT = Sydney Language Battery; WM = white matter.

|  | **Factor 1: Brain & Vascular Health** | | **Factor 2: Structural Integrity** | | **Factor 3: WM Fluid Dysregulation** | | **Factor 4: AD Biomarkers** | | **Factor 5: Neuronal Injury** | |
| --- | --- | --- | --- | --- | --- | --- | --- | --- | --- | --- |
|  | β (SE) | *p* | β (SE) | *p* | β (SE) | *p* | β (SE) | *p* | β (SE) | *p* |
| **Prose Passages Delayed** | 0.150 (0.140) | .287 | 0.091 (0.093) | .327 | -0.015 (0.098) | .878 | -0.059 (0.093) | .527 | -0.185 (0.097) | .060 |
| **Similarities** | 0.240 (0.135) | .073 | -0.037 (0.089) | .676 | -0.089 (0.094) | .345 | -0.119 (0.089) | .182 | **-0.219 (0.092)** | **.019** |
| **SYDBAT Naming** | 0.141 (0.139) | .312 | 0.016 (0.092) | .865 | -0.066 (0.097) | .498 | 0.024 (0.093) | .798 | -0.035 (0.098) | .722 |
| **Visual Reproduction Delayed** | 0.074 (0.134) | .583 | 0.093 (0.088) | .296 | 0.047 (0.094) | .617 | -0.040 (0.089) | .654 | **-0.188 (0.093)** | **.045** |
| **Trail Making  Test B*** | -0.212 (0.132) | .111 | 0.002 (0.088) | .983 | -0.025 (0.093) | .790 | -0.084 (0.088) | .343 | 0.159 (0.092) | .087 |
| **Verbal Fluency** | 0.255 (0.135) | .061 | 0.046 (0.090) | .614 | -0.083 (0.095) | .383 | -0.036 (0.091) | .692 | -0.054 (0.096) | .570 |
| **Hooper Visual Organisation Test** | -0.042 (0.140) | .766 | 0.074 (0.092) | .425 | 0.042 (0.098) | .664 | 0.141 (0.092) | .129 | -0.033 (0.098) | .737 |

**References:**

1. Esteban O, Birman D, Schaer M, et al. MRIQC: Advancing the automatic prediction of image quality in MRI from unseen sites. Bernhardt BC. ed. PLOS ONE 2017;12(9):e0184661; doi: 10.1371/journal.pone.0184661.

2. Gorgolewski K, Burns CD, Madison C, et al. Nipype: A Flexible, Lightweight and Extensible Neuroimaging Data Processing Framework in Python. Front Neuroinformatics 2011;5; doi: 10.3389/fninf.2011.00013.

3. Chen JJ, Rosas HD, Salat DH. Age-associated reductions in cerebral blood flow are independent from regional atrophy. NeuroImage 2011;55(2):468–478; doi: 10.1016/j.neuroimage.2010.12.032.

4. Shao X, Ma SJ, Casey M, et al. Mapping water exchange across the blood-brain barrier using 3D diffusion-prepared arterial spin labeled perfusion MRI. Magn Reson Med 2019;81(5):3065–3079; doi: 10.1002/mrm.27632.

5. Shao X, Jann K, Ma SJ, et al. Comparison Between Blood-Brain Barrier Water Exchange Rate and Permeability to Gadolinium-Based Contrast Agent in an Elderly Cohort. Front Neurosci 2020;14:571480; doi: 10.3389/fnins.2020.571480.

6. Shao X, Shou Q, Felix K, et al. Age-Related Decline in BBB Function Is More Pronounced in Males than Females. 2024; doi: 10.7554/eLife.96155.1.

7. Lynch KM, Sepehrband F, Toga AW, et al. Brain perivascular space imaging across the human lifespan. NeuroImage 2023;271:120009; doi: 10.1016/j.neuroimage.2023.120009.

8. Menze I, Bernal J, Kaya P, et al. Perivascular space enlargement accelerates in ageing and Alzheimer’s disease pathology: evidence from a three-year longitudinal multicentre study. Alzheimers Res Ther 2024;16(1):242; doi: 10.1186/s13195-024-01603-8.
